# Multi-criteria decision-making for prioritizing photocatalytic processes followed by TiO_2_-MIL-53(Fe) characterization and application for diazinon removal

**DOI:** 10.1038/s41598-023-34306-5

**Published:** 2023-05-01

**Authors:** Fateme Barjasteh-Askari, Ramin Nabizadeh, Aliasghar Najafpoor, Mojtaba Davoudi, Amir-Hossein Mahvi

**Affiliations:** 1 Department of Environmental Health Engineering, School of Public Health, Tehran University of Medical Sciences, PourSina St., Qods St., Enghelab St., Tehran, Iran; 2 Department of Environmental Health Engineering, School of Health, Torbat Heydariyeh University of Medical Sciences, Torbat Heydariyeh, Iran; 3 Health Sciences Research Center, Torbat Heydariyeh University of Medical Sciences, Torbat Heydariyeh, Iran; 4 Center for Air Pollution Research (CAPR), Institute for Environmental Research (IER), Tehran University of Medical Sciences, Tehran, Iran; 5 Social Determinants of Health Research Center, Mashhad University of Medical Sciences, Mashhad, Iran; 6 Department of Environmental Health Engineering, School of Health, Mashhad University of Medical Sciences, Mashhad, Iran; 7 Center for Solid Waste Research, Institute for Environmental Research, Tehran University of Medical Sciences, Tehran, Iran

**Keywords:** Environmental sciences, Catalysis

## Abstract

Multi-criteria decision-making (MCDM) can introduce the best option based on evidence. We integrated the Analytic Hierarchy Process (AHP) and the Technique for Order of Preference by Similarity to the Ideal Solution (TOPSIS) to prioritize the alternatives for photocatalytic diazinon removal in a bench scale and characterized TiO_2_-MIL-53(Fe) for this purpose. Criteria and alternatives were listed based on systematic literature reviews and expert opinions. Then, AHP and TOPSIS questionnaires were developed and distributed to an expert panel for pairwise comparisons. We converted the linguistic variables into the corresponding fuzzy values and used R for mathematical calculations. Then, TiO_2_-MIL-53(Fe) was synthesized and characterized for diazinon removal under LED visible light. The AHP ranked criteria as availability > degradation efficiency > safety for the environment > material cost > energy consumption > mineralization efficiency > photocatalyst reusability > safety for personnel > equipment cost. Based on TOPSIS, the order of alternatives was TiO_2_-containing/Visible light > ZnO-containing/UV light > TiO_2_-containing/UV light > ZnO-containing/Visible light > WO_3_-containing/UV light. With a bandgap of 1.8 eV, TiO_2_-MIL-53(Fe) could remove 89.35% of diazinon at 10 mg/L diazinon concentration, 750 mg/L catalyst dose, pH 6.8, and 180-min reaction time. Hybrid AHP-TOPSIS identified the best option for photocatalytic diazinon removal from aqueous solutions. Thus, MCDM techniques can use systematic review results to overcome the uncertainty in designing experimental studies.

## Introduction

Diazinon is a widely used organophosphorus pesticide categorized into class II chemicals (probably carcinogenic to humans) by the World Health Organization (WHO)^1^. It is a non-systemic, contact, digestive, and respiratory toxicant inhibiting the acetylcholinesterase enzyme of pests irreversibly, leading to central nervous system impairment. It is applied primarily in agricultural fields to warrant decreased losses of crops and increased productivity to meet the ever-increasing needs of growing populations worldwide^2^. However, its emergence through various routes, mainly agricultural runoff, harms the environment, including aquatic and terrestrial life^3,4^. It also deteriorates the quality of surface and groundwater resources, necessitating advanced technologies for drinking water treatment^5,6^.

Numerous treatment technologies can be practiced for diazinon removal, including adsorption^7^, electrocoagulation^8^, biological processes^9^, membrane technologies^10^, sonochemical degradation^11^, and Advanced Oxidation Processes (AOPs), including photocatalytic processes^12^. Photocatalytic processes to remove organic pollutants usually require a semiconductor as the photocatalyst to be excited by irradiation under visible or ultraviolet light^13^. There are different photocatalysts, such as metal oxides (ZnO, TiO_2_, WO_3_, CeO_2_, Fe_2_O_3_), metal sulfides (CdS, PbS, CuS), Metal–Organic Frameworks (MOFs), and polymer-based materials, which can be used in either pristine or modified forms to accelerate photo-driven reactions^14^. When a photocatalyst is exposed to light having enough energy to overcome the photocatalyst band gap energy, an electron moves from the valence band to the conduction band, leading to the generation of electron–hole pairs^15^. Organic pollutants are directly oxidized through these positive holes. Moreover, the interaction between the hole and the water molecules and soluble ions generates highly reactive radicals, most importantly ^•^OH, which have the primary role in decomposing organic pollutants^16,17^.

There are many role players in photocatalytic systems, which may confuse researchers and hinder straightforward decision-making. They may include process efficiency, costs (capital, operation, and maintenance), safety, and robustness^18^, besides photocatalyst availability, reusability, and stability^19^. To be specific, TiO_2_ is an available and relatively low-cost photocatalyst that efficiently removes diazinon^14^, but due to its wide band gap, it is only excited in the UV spectrum^20^, increasing energy costs and raising safety concerns. The MOFs are newly emerging photocatalysts that can be activated in the visible spectrum^21^; however, they are complicated and produced with toxic precursors such as DMF. On the other hand, local conditions, such as the technology level, skilled labor force, and economic status, can also influence the final choice of the best available technology in fieldwork^22^, which may vary across countries. According to Collivignarelli, et al.^23^, challenges in finding a suitable photocatalyst, costs of purchasing photocatalysts and operating the system, and technical complexities such as the rapid recombination of electron–hole pairs limit the large application of photocatalysis. Therefore, we must use a comprehensive decision-making system to select the most advantageous system. In this regard, Multi-Criteria Decision-Making (MCDM) techniques as normative models can help identify the best alternative among various options^24^. The main advantage of MCDM techniques is that they enable us to select the best alternative based on multiple criteria from different perspectives, not just one aspect, e.g., removal efficiency.

The fuzzy AHP-TOPSIS method is a hybrid solution for solving the problem based on Multi-Criteria Decision-Making (MCDM) principles. The fuzzy Analytic Hierarchy Process (AHP) works in a hierarchical framework in which the rater determines a weight for each criterion involved in selecting alternatives. The theoretical framework of fuzzy AHP is fully presented by Hsieh et al.^25^. The Technique for Order of Preference by Similarity to the Ideal Solution (TOPSIS) gives priority to an alternative based on similarity to the ideal solution. It also necessitates some calculations, as Alptekin et al.^26^ described. Although each method is robust for decision-making, the hybrid technique can overcome each method's flaws. In the first step, the selected criteria are weighted using the fuzzy AHP to find the relative criteria preferences. In the next step, these weights are used in fuzzy TOPSIS to rank the alternatives based on the distances from Positive Ideal Solution (PIS) and Negative Ideal Solution (NIS). Based on a study by Mardani et al.^27^, the AHP was the most practiced individual tool, and hybrid MCDM was the most frequently applied integrated method for decision-making in a systematic review of studies published between 2000 and 2014.

In a previous study^14^, we employed a systematic literature review to explore the current knowledge about various aspects of a photocatalytic system for diazinon removal from aqueous solutions. The extracted data are used in the current study as the input to implement the fuzzy AHP-TOPSIS and rank the available options in the literature. The goal was to select the best applicable process based on evidence to organize a bench-scale study for photocatalytic diazinon removal. Then, we fabricated a photocatalyst based on the obtained results and used it for diazinon removal in a preliminary experimental study. To the best of our knowledge, it is the first study that introduces MCDM techniques in the photocatalysis domain.

## Materials and methods

This study was approved by the Research Ethics Committee of the Faculty of Health, Tehran University of Medical Sciences, Tehran, Iran, and followed the relevant guidelines and regulations. It was conducted in two phases. In phase I, an integrated AHP-TOPSIS was implemented to disclose the best photocatalytic system for diazinon removal. In phase II, experiments were carried out to determine the characteristics of the select photocatalyst and the efficacy of the photocatalytic system for diazinon removal.

### Criteria and alternatives selection

Two authors (FBA and MD) conducted an extensive literature search to provide the research team with a preliminary list of criteria concerning the technical and managerial aspects of bench-scale photocatalysis. They had a history of dealing with the literature on photocatalytic diazinon removal, leading to a published systematic review^14^, implying their competencies. However, the research team was free to enrich the list with additional criteria based on their knowledge and experience. Finally, six criteria were selected based on the research team's opinions and the literature^18,19,23^, as follows: Process efficiency (sub-criteria: degradation efficiency and mineralization efficiency), process cost (sub-criteria: material cost and equipment cost), availability, photocatalyst reusability, energy consumption, and process safety (sub-criteria: personnel and the environment). These criteria were employed to appraise the most widely used photocatalytic systems in the experimental studies retrieved from our systematic review of 777 articles published in the recent 5 years with a sound methodology described elsewhere^14^. We categorized the alternatives based on the photocatalyst and light source as follows: TiO_2_-containing/Visible light, TiO_2_-containing/UV light, ZnO-containing/Visible light, ZnO-containing/UV light, and WO_3_-containing/UV light. Finally, a hierarchy diagram was drawn according to the criteria/sub-criteria and alternatives, as shown in Fig. S1.

### Expert panel establishment

Expert panels are widely used for weighing criteria and evaluating alternatives in MCDM techniques^28^. The expert panel in this study included 18 Ph.D. holders in Environmental Health Engineering with an experience in photocatalytic removal of persistent pollutants. They were purposefully selected from different universities across Iran. Due to COVID-19 limitations, they were contacted via emails to be briefed about the study procedure and provided with AHP and TOPSIS questionnaires. Giving informed consent to participate was a prerequisite for recruiting panel members.

### Fuzzy AHP running

A questionnaire was designed for running AHP in three parts. The first part introduced the objectives, criteria, and the procedure to the expert panel. The second part comprised the pairwise comparisons of criteria and sub-criteria. In this part, each criterion/sub-criterion was compared with its pairs concerning their importance in selecting an alternative for the photocatalytic removal of diazinon in the bench scale. The third part included a fact sheet derived from reliable literature, presenting the current knowledge about criteria and sub-criteria to help the expert panel fill out the questionnaire. To do so, the expert panel members applied linguistic variables that were then converted into their corresponding fuzzy values based on the Delphi method^29^, as shown in Table S1. Next, a matrix was developed for each completed questionnaire. The obtained matrices were checked for consistency of judgments based on the method presented by Mohamed Salah-eldin^30^. In this method, an inconsistency index was calculated for each matrix. Consistent matrices were those with an inconsistency index of ≤ 0.1 and were utilized to calculate the fuzzy weight of each criterion/sub-criterion through the geometric mean method using fuzzy AHP package version 0.9.0 in R software. Then, the Best Non-Fuzzy Performance (BNP) values were computed to rank the criteria. At the end of the AHP, a weight was assigned to each criterion/sub-criterion and used for fuzzy TOPSIS calculations.

### Fuzzy TOPSIS running

In this step, another questionnaire was designed to ask the experts' opinions about the available alternatives relative to each other concerning each criterion/sub-criterion. The first part explained the purpose of the questionnaire and its procedure. Besides, the raters were provided with a fact sheet about alternatives respecting the criteria. This information was obtained from a literature search, as described elsewhere^14^. In the next part, a table of alternatives versus criteria/sub-criteria was developed, and the expert panel was asked to use linguistic variables to rate each alternative relative to others (Table S2). Only experts with consistent AHP matrices were invited to fill out the TOPSIS questionnaires in this step. The experts were trained to value the alternatives based on their knowledge, experience, and the literature data we provided as the fact sheet. Besides, they were instructed to judge only the given catalysts as pristine regardless of various, endless modifications applicable to change the photocatalyst characteristics for various reasons.

The linguistic variables were converted into fuzzy values in the next step, and a fuzzy matrix was created for each questionnaire. All matrices were integrated into a unit matrix, called the "aggregate fuzzy decision matrix." The aggregate matrix was normalized in the next step to rescale the values from 0 to 1. For normalization, each fuzzy value in a triangular fuzzy set was divided by the maximum upper bound value for each criterion, as described elsewhere^26^. Then, all normalized fuzzy values in the matrix were multiplied by their fuzzy weights attained from the fuzzy AHP. Therefore, a weighted normalized fuzzy decision matrix was created. Next, the Fuzzy Positive Ideal Solution (FPIS) and Fuzzy Negative Ideal Solution (FNIS) were calculated to quantify the distance of each alternative from the ideal solutions represented as positive and negative ideal solutions (d^+^ and d^−^, respectively). Finally, the Closeness Coefficient (CCi) and Normalized CCi (NCCi) were calculated for each alternative and used for ranking. Indeed, the CCi measured the distance of each alternative from FPIS. The best alternative had the highest CCi and an NCCi closer to unity^31^.

### Experimentation

The select photocatalytic process from the AHP-TOPSIS technique, i.e., TiO_2_-containing/Visible light, was practiced for diazinon removal from aqueous solutions. We fabricated a TiO_2_-MIL-53(Fe) composite, which has never been employed for diazinon removal, by combining the methods described by Zhang et al.^32^ and Zhang et al.^33^ to use in the presence of LED visible light. Briefly, 170 mg TiO_2_ (anatase, Merck, Germany) was added to the mixture of 24 mL DMF (99.8%, Merck, Germany) and 2 mL absolute ethanol (Romil, UK). After 30 min ultrasonication (Elm sonic S30H, Elma Schmidbauer GmbH, Singen, Germany), 0.332 g H_2_BDC (98%, Merck, Germany) and 0.27 g FeCl_3_·6H_2_O (Merck, Germany) were added under vigorous mixing for 30 min. Then, the whole mixture was placed in a Teflon-lined stainless-steel autoclave (60 mL) at 150 °C for 15 h. The product was washed several times using DMF and ethanol and dried in 150 °C for 12 h.

A Field Emission Scanning Electron Microscopy device (FESEM; TESCAN BRNO-Mira3 LMU, Czech Republic) coupled with energy dispersive X-ray spectroscopy (EDS) determined the photocatalyst's morphology and elemental composition. A UV–visible spectrophotometer (Shimadzu UV-160A, Japan) was utilized to determine the optical properties of the photocatalyst in a wide range of wavelengths. The data were used to draw Tauc plots and calculate the band gap of the photocatalysts. Photoluminescence (PL) spectra were used to determine the recombination rate using a Varian Cary Eclipse Fluorescence Spectrophotometer (Agilent Co., USA) equipped with a xenon lamp at the excitation wavelength of 300 nm. Moreover, Mott–Schottky analysis was performed at a 500 Hz frequency in 0.1 M Na_2_SO_4_ solution (pH = 6.8) to determine the flat band potential and shed light on reaction mechanisms. The X-ray diffraction analysis was conducted to determine the structure of the fresh and recycled photocatalyst in a 2θ range of 1°–80° (XRD Explorer, GNR, Italy; Dectris detector; Tube Cu Kα = 1.54 Å, Voltage 40 kV, and Current 30 mA).

The photocatalyst experiments were conducted at a photocatalyst dose of 750 mg/L, diazinon concentration of 10 mg/L, solution pH of 6.8, and irradiation duration of 180 min. A 30-min reaction time was allowed in the dark before irradiation to compensate for the adsorption effect on photocatalysis efficacy. Adsorption and photolysis experiments were performed for the same duration without light and photocatalyst, respectively. The photocatalyst was employed in three successive experiments after regeneration for reusability assessment. Then, removal efficiencies were compared across the cycles.

A batch reactor made of glass was utilized with a total volume of 100 mL. A 50 mL working volume was employed and constantly agitated at 250 rpm during reactions on a magnetic stirrer. Irradiation was performed with two COB LED lamps (50 W power each) emitting visible light above 380 nm. Each lamp was fixed at a distance of 1 cm from each side of the vessel. The diazinon concentration in the initial and final solutions was determined with an HPLC apparatus (Knauer Smartline, Germany) equipped with a UV/Visible detector (set at 250 nm) and a separation column (C18, 150 mm × 4.6 mm). The methanol-to-water volumetric ratio of 80/20 at a flow rate of 1.2 ml/min was used for the mobile phase. The removal efficiency was computed using Eq. (1):

1 $$ R = \frac{{C_{i} - C_{f} }}{{C_{i} }} \times 100 $$

where *R* is the removal efficiency (%), *C*_*i*_ is the initial diazinon concentration (mg/L), and *C*_*f*_ is the final diazinon concentration (mg/L).

### Ethical approval

This study was approved by the Research Ethics Committee of the Faculty of Health, Tehran University of Medical Sciences (IR.TUMS.SPH.REC.1400.196).

### Informed consent

All the panel members voluntarily filled out AHP-TOPSIS questionnaires and gave their informed consent to participate.

## Results and discussion

We identified six criteria for the appraisal of bench-scale photocatalytic techniques, including process efficiency (degradation and mineralization), process cost (material and equipment), availability, photocatalyst reusability, energy consumption, and safety (personnel and the environment). The alternatives found in the literature for photocatalytic diazinon removal were TiO_2_-containing/Visible light, TiO_2_-containing/UV light, ZnO-containing/Visible light, ZnO-containing/UV light, and WO_3_-containing/UV light.

### Fuzzy AHP-TOPSIS results

An expert panel of 18 members was established to make pairwise comparisons between criteria/sub-criteria using linguistic variables. Then, a matrix was developed for each response (n = 18), with inconsistency indices of 0.05–0.4. After removing inconsistent matrices (n = 7), the remaining ones (n = 11) with ≤ 0.1 inconsistency indices were used for calculating the fuzzy weight of criteria/sub-criteria through the geometric mean method. This method generated the integrated matrices for criteria (Table S3) and sub-criteria (Table S4). The integrated matrix of criteria had a consistency ratio of 0.005. Table 1 shows that the BNP fractions were 0.231 for efficiency, 0.208 for safety, 0.201 for costs, 0.150 for availability, 0.120 for energy consumption, and 0.083 for photocatalyst reusability. Concerning sub-criteria, degradation efficiency was superior to mineralization efficiency, material cost to equipment cost, and environmental safety to personnel safety. Respecting the individual BNP values, the order of importance was as follows: Availability > degradation efficiency > safety for the environment > material cost > energy consumption > mineralization efficiency > photocatalyst reusability > safety for personnel > equipment cost. In a similar study, Azari et al.^34^ utilized integrated fuzzy AHP-TOPSIS for prioritizing dye removal processes using carbon-based adsorbents. They showed accessibility as the most important appraisal criterion, followed by reusability, adsorption capacity, environmental safety, human safety, material cost, and equipment cost, with BNP values in the range of 0.049–0.250.

**Table 1:** AHP weights of criteria for the appraisal of bench-scale photocatalytic techniques.

Criteria	Sub-criteria	Weights			BNP	Ranking
		L	M	U		
Efficiency	Degradation efficiency	0.078	0.134	0.202	0.132	2
	Mineralization efficiency	0.0545	0.100	0.164	0.099	6
Cost	Material cost	0.073	0.123	0.188	0.122	4
	Equipment cost	0.043	0.080	0.134	0.079	9
Availability	–	0.109	0.150	0.199	0.150	1
Photocatalyst reusability	–	0.0595	0.082	0.115	0.083	7
Energy consumption	–	0.084	0.119	0.167	0.120	5
Safety	Safety for personnel	0.051	0.082	0.124	0.081	8
	Safety for the environment	0.085	0.130	0.182	0.127	3

The criteria weights emerging from the fuzzy AHP were used to complete the fuzzy TOPSIS. Eleven eligible experts completed the TOPSIS questionnaires. The aggregate fuzzy decision matrix is given in Table S5, and its normalized matrix is shown in Table S6. A weighted normalized fuzzy decision matrix was created after applying the criteria weights obtained from fuzzy AHP, as summarized in Table S7. Then, the distances from positive and negative ideal solutions (d^+^ and d^−^, respectively) were measured for each alternative, followed by CCi and NCCi calculations. Table 2 demonstrates the final results of fuzzy TOPSIS and the ranking of alternatives based on NCCi. As can be seen, the preference order of the alternatives was as follows: TiO_2_-containing/Visible light > ZnO-containing/UV light > TiO_2_-containing/UV light > ZnO-containing/Visible light > WO3-containing/UV light. Therefore, in the framework of this study, we identified TiO_2_-containing/Visible light as the best photocatalysis system for diazinon removal. Based on the NCCi values of 0.1124–0.1395, Azari et al.^34^ ranked carbon-based adsorbents for dye removal as follows: powdered activated carbon > granular activated carbon > carbon nanotubes > graphene oxide > graphene > reduced graphene oxide > graphite > coal.

**Table 2:** Indices and ranking of diazinon photocatalytic removal based on fuzzy TOPSIS.

Alternative	d^+^	d^−^	CCi	NCCi	Ranking
TiO_2_-containing/UV	0.151	0.213	0.585	0.193	3
TiO_2_-containing/Vis	0.065	0.253	0.796	0.262	1
ZnO-containing/UV	0.137	0.262	0.657	0.216	2
ZnO-containing/Vis	0.194	0.222	0.533	0.175	4
WO_3_-containing/UV	0.220	0.196	0.472	0.155	5

With a molecular weight of 79.87 g/mol, TiO_2_ is a traditional photocatalyst used in the experimental removal of persistent pollutants. It is a readily available material with an easy application^35^. Thus, it gains a high score on availability. However, TiO_2_ has a wide band gap (3.2 eV, as obtained in this study), making it only active at wavelengths below 385 nm^36^. Therefore, it must be modified before applying in the visible light spectrum. Studies have shown that modified TiO_2_ can be highly efficient under visible light and completely remove diazinon. For example, Nakaoka et al.^37^ utilized Pt-doped TiO_2_ in the presence of a 900 W xenon lamp and achieved 100% diazinon removal. Zangeneh et al.^38^ and Molla et al.^39^ also reported comparable results.

Concerning safety for the environment, photocatalysts used for water and wastewater technologies are often inert nanoparticles that impose no extra hazard to the environment; besides, they must be safe for biological systems and have low toxicity^40–42^. TiO_2_ is no exception; its 96 h-LC50 has been measured as > 1000 mg/L for fish, and its 48 h-EC50 has been reported as > 1000 mg/L for *Daphnia magna*. According to the TiO_2_ Material Safety Data Sheet in the literature, it is also readily degradable in the environment. Besides, TiO_2_ can be prepared at a low cost, as titanium is naturally found in the earth's crust with an abundance of 0.44%^43^. In the laboratory, it can be produced by several facile methods, including hydrothermal, solvothermal, sol–gel, chemical precipitation, electrodeposition, direct oxidation, sonochemical, and microwave methods^44^. These methods necessitate the use of precursors such as titanium tetra-ethoxide Ti(OEt)_4_, titanium tetra-isopropoxide Ti(OPr^i^)_4_, titanium ethoxide Ti(OC_2_H_5_), titanium isopropoxide Ti(OC_3_H_7_)_4_, titanyl sulfate TiOSO_4_, titanium tetrachloride TiCl_4_, and titanium trichloride TiCl_3_^45^. Moreover, quality-grade TiO_2_ powder is readily available for laboratory experiments from Sigma Aldrich and Merck suppliers with reasonable costs for research purposes.

Energy consumption in photocatalysis largely depends on the characteristics of the light source for irradiation, including electrical current intensity and working voltage, critical factors for operating costs. The Electrical Energy per Order (E_EO_) is usually expressed as a measure of energy consumption in photocatalytic processes to tradeoff between cost and effectiveness. E_EO_ is the amount of electrical energy (kWh) needed for one order of magnitude reduction of pollutant concentration in the unit volume of solution. Therefore, it is proportional to the power of the light source and the irradiation duration to reach a 90% removal rate. Although the E_EO_ of various photocatalytic technologies was reported from 38.93^46^ to 350.36 kWh/m^3^ ^47^, the literature lacked this data concerning diazinon removal with TiO_2_-containing/Visible light, which needs to be elucidated in future studies.

The mineralization efficiency concerns the complete oxidation of organic carbon to inorganic carbon, CO_2_. Therefore, it requires measuring the total organic content of the aqueous solution before and after photocatalysis. A mineralization efficiency close to 100% indicates fewer organic by-products to be concerned about, as research indicated that some by-products are more toxic than their parental compounds^48^. The mineralization reaction for diazinon mineralization is proposed as follows^37^:

2 $$ C_{12} H_{21} N_{2} O_{3} PS + 21O_{2} \to 2HNO_{3} + H_{3} PO_{4} + H_{2} SO_{4} + 12CO_{2} + 7H_{2} O $$

The literature has reported the mineralization efficiency of TiO_2_-containing/Visible light processes between 63^49^ and 100%^38^. The latter was obtained in the presence of B/TiO_2_-SiO_2_/CoFe_2_O_4_ as the catalyst and 18 W fluorescent lamps as the light source.

The photocatalyst reusability can be a significant factor in the economy of photocatalysis, as the photocatalyst is usually a precious material and is required in large amounts of up to 600 mg/L in optimum conditions^14^. In experiments, reusability is measured by successively using the regenerated photocatalyst under the same experimental conditions and then comparing the removal efficiencies across the cycles. The regenerated photocatalyst should show an efficiency close to that of the fresh one. TiO_2_ catalysts are usually accompanied by good reusability characteristics. A study^50^ incorporated TiO_2_ into palladium heterogeneous catalysts to improve its reusability. Reusability can also pinpoint the stability feature, as unstable photocatalysts can release their ingredients into the solution, leading to secondary contamination. Salarian et al.^49^ and Zangeneh et al.^38^ claimed negligible decreases in diazinon removal efficiency after seven and three cycles of TiO_2_-containing/Visible light photocatalysis from 85 to 80% and 100 to 96%, respectively.

Concerning personnel safety, working in the laboratory cannot be without risks to the staff and examiners. In photocatalysis, the risks may root in contact with chemicals and exposure to light. Based on the United Nations' Globally Harmonized System (GHS), TiO_2_ is a probable carcinogen, placed in category 2 of carcinogens. Therefore, it must be avoided from any contact with any concentration. However, USEPA recognizes it as a source of low concern based on experimental and modeled studies^51^. Photocatalytic systems utilizing UV radiation pose additional risks to people, as UV exerts high-energy photons harmful to body organs, including the eyes and skin. Ultraviolet is a probable carcinogen to the skin^52^ and an eye irritant^53^, depending on exposure duration, light intensity and wavelength, and distance from the source^54^. Therefore, concerning safety regulations, low-energy visible light might be advantageous over UV light. However, recent research has postulated a role for high-intensity visible light, as is usual for photocatalysis, in skin damage symptoms such as hyperpigmentation^55^.

Here, we referred to the light source costs as the equipment cost for photocatalysis to distinguish between UV and visible light sources. This criterion had the least relevance to selecting photocatalytic processes in our study. Lamps are available at various prices depending on their light-emitting mechanisms. Costly mercury lamps are the traditional options for UV light generation, while sunlight is free for having visible light necessary for activating the photocatalyst. In recent years, the advancement in Light-Emitting Diodes (LEDs) technology has made them attractive for irradiation. LED lamps need small installation spaces, have high energy efficiencies, and are much less costly. Although they can emit a broad light spectrum from UV to visible, they have been less utilized for photocatalysis than mercury lamps for UV light and xenon lamps for visible light. Owing to their unique characteristics, they can open a new window for researchers to remove persistent pollutants more cost-effectively.

### Characteristics of the select photocatalyst

**Figure 1:**
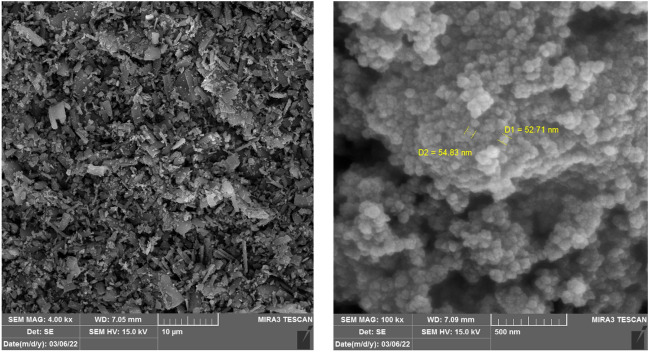
SEM images of TiO_2_-MIL-53(Fe) at 4X (left) and 100X (right) magnifications.

Based on the fuzzy AHP-TOPSIS results and in the framework of this study, the TiO_2_-containing/Visible light photocatalytic process emerged as the best alternative for diazinon removal from aqueous solutions. Therefore, this process was utilized in a set of experiments to determine its efficiency. As known, TiO_2_ is a semiconductor that needs modification to be activated in visible light. In this study, we fabricated a TiO_2_-MIL-53(Fe) composite. Figure 1 demonstrates the SEM images of TiO_2_-MIL-53(Fe) at different magnifications to present the morphology of the as-prepared photocatalyst. Based on the image, TiO_2_ nanoparticles were successfully incorporated into the metal–organic framework structure. TiO_2_ nanoparticles surrounded the rod-like MIL-53(Fe) particles to make a rough surface. Figure S2 demonstrates the EDS plots and compositional analysis of TiO_2_, MIL-53(Fe), and TiO_2_-MIL-53(Fe). As can be seen, the composite consisted of Ti (18.90%), C (37.18%), O (41.78%), and Fe (2.14%). These results corroborate the findings of Zhang et al.^33^, in which the synthesized MIL-53(Fe) gradually lost its polyhedron shape as it was incorporated with different amounts of TiO_2_. In the EDS spectra, they detected C, O, Fe, and Ti elements at percentages proportional to the added amounts of TiO_2_ precursor.

**Figure 2:**
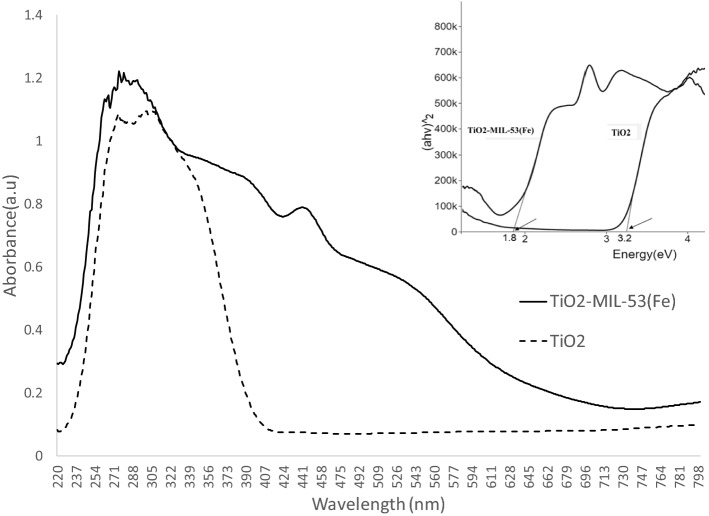
UV–visible spectroscopy of photocatalysts; the inset shows the Tauc plots, indicating the band gaps.

Figure 2 shows the UV–Vis spectra of TiO_2_ and TiO_2_-MIL-53(Fe), confirming TiO_2_-MIL-53(Fe) as a photocatalyst. The figure inset shows the corresponding Tauc plots, indicating the band gap energy of TiO_2_ at 3.2 eV and TiO_2_-MIL-53(Fe) at 1.8 eV. The TiO_2_-MIL-53(Fe) narrow band gap implies its potential for activation in the visible light spectrum. In other words, it could be activated at a wavelength of 680 nm. These results align with the Zhang, et al.^33^ study, in which MIL-53(Fe)@TiO_2_ showed a bandgap of 1.98 eV in the optimum fabrication condition.

**Figure 3:**
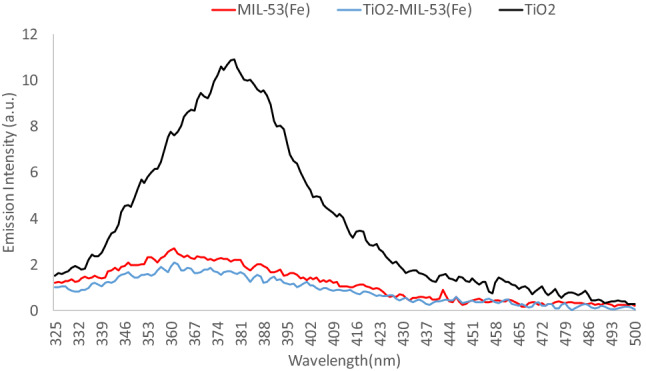
PL spectra of TiO_2_, MIL-53(Fe), and TiO_2_-MIL-53(Fe).

PL spectra in Fig. 3 show a large peak for TiO_2_, indicating a high electron–hole recombination rate. The difference between the peak intensity of MIL-53(Fe) and TiO_2_-MIL-53(Fe) shows the longer life of electron–hole pairs on the modified photocatalyst. So, it can be inferred that TiO_2_-MIL-53-(Fe) composite has a higher separation efficiency and charge lifetime^33,56^.

**Figure 4:**
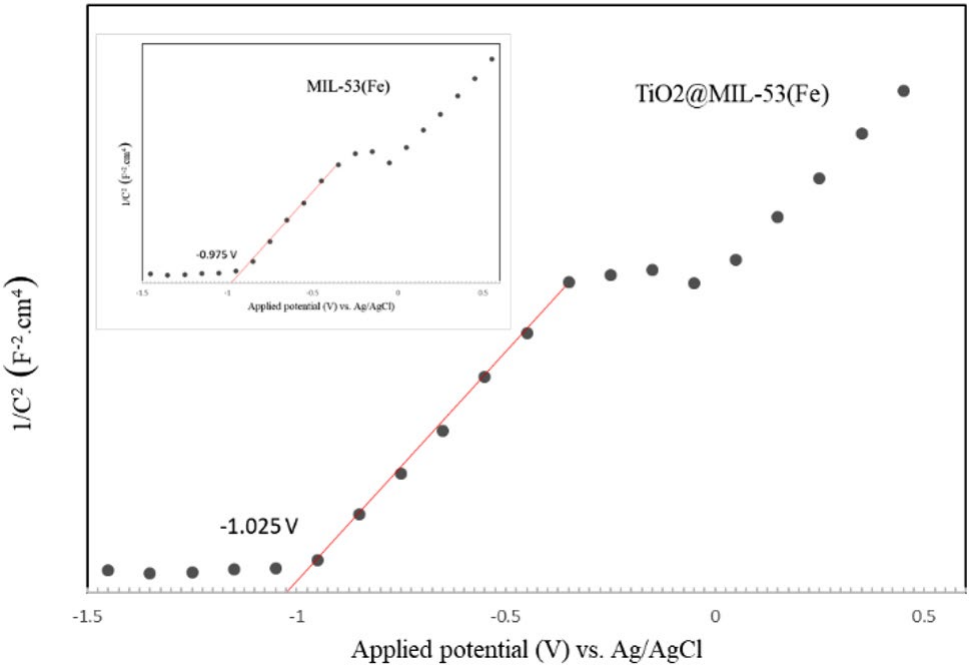
Mott–Schottky plots of TiO_2_-MIL-53(Fe) and MIL-53(Fe) photocatalysts.

**Figure 5:**
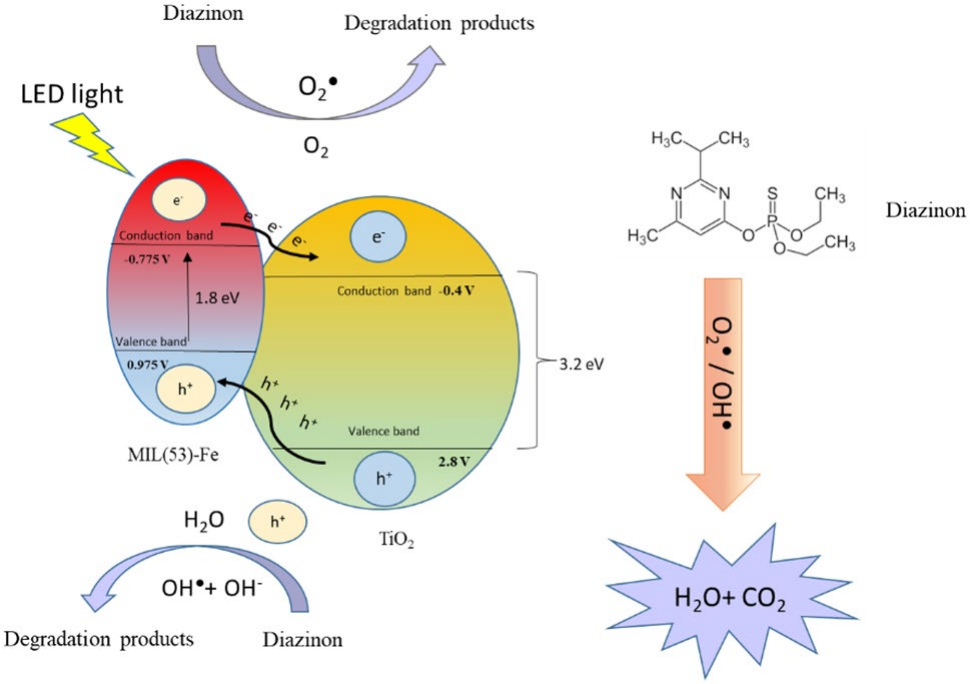
The activation mechanism and charge transfer route of TiO_2_-MIL-53(Fe) photocatalyst.

Figure 4 demonstrates the Mott–Schottky plots of TiO_2_-MIL-53(Fe) and MIL-53(Fe). The flat band potential (V_fb_) was measured using this graph. As can be seen, both photocatalysts are n-type due to the positive slope of the curves^56^. In the case of TiO_2_-MIL-53(Fe), the conduction band potential (E_CB_) and valance band potential (E_VB_) are calculated as − 0.825 V and 0.975 V versus NHE, respectively. The V_fb_ of MIL-53(Fe) is − 0.975 V versus Ag/AgCl at pH 6.8. It should be noted that the conduction band in n-type photocatalysts is very close to V_fb_. Therefore, E_CB_ is − 0.775 V versus NHE. On the other hand, E_VB_ can be calculated through the difference between the band gap value (E_g_) and E_CB_^33^. Thus, regarding the MIL-53(Fe) band gap, which was measured as 1.8 eV in this study, E_VB_ is calculated as 0.975 V versus NHE. As shown earlier, the band gap of TiO_2_ was 3.2 eV. Moreover, according to the literature^57^, E_CB_ and E_VB_ of TiO_2_ are − 0.4 V and 2.8 V versus NHE, respectively. Due to the position of CB in MIL-53(Fe) and TiO_2_, photogenerated electrons can transfer onto the CB of TiO_2_. On the other hand, positive holes produced on TiO_2_ can migrate onto the VB of MIL-53(Fe), reducing recombination and improving efficiency^33,56,57^. The obtained data were utilized to elucidate the mechanism of photocatalyst activation. Figure 5 demonstrates the graphical representations of the activation mechanism and charge transfer route of TiO_2_-MIL-53(Fe).

### Experimental results

**Figure 6:**
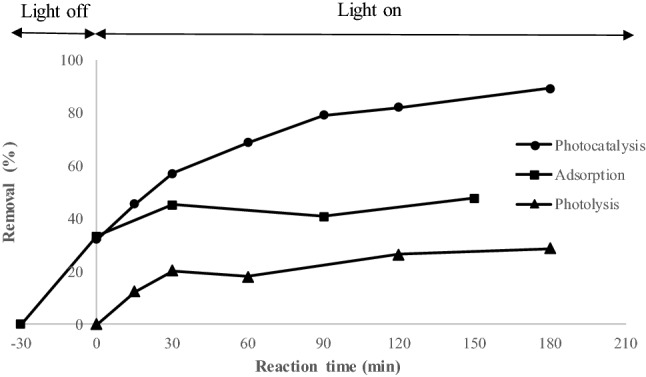
The experimental results of diazinon removal using photocatalysis, adsorption, and photolysis; TiO_2_-MIL-53(Fe) was used as adsorbent and photocatalyst.

Figure 6 depicts the experimental results of diazinon removal using TiO_2_-MIL-53(Fe). These results proved the efficacy of TiO_2_-MIL-53(Fe)/Visible light for diazinon abatement. The results showed that the as-prepared catalyst could remove diazinon via adsorption and oxidation mechanisms. However, its adsorption efficiency was limited, so it maximally reached 47.82% after 180 min contact time. On the other hand, irradiation under visible light drastically increased the removal rate, so a maximum removal of 89.35% was achieved after 180 min. It seems that adsorption was the dominant mechanism at the initial reaction times. However, the occupation of active sites with diazinon molecules made this mechanism inefficient at later times. TiO_2_-MIL-53(Fe) activation under visible light reinforced the removal rate by generating reactive oxygen species, which effectively participate in the degradation of diazinon. The photolysis experiments without TiO_2_-MIL-53(Fe) showed a removal rate of 28.58% after 180 min. As the removal rate of photocatalysis is higher than the sum of adsorption and photolysis removal rates (89.53% > 47.82% + 28.58%), it shows the synergistic effect of photocatalysis for diazinon removal. Zhang et al.^33^ demonstrated that MIL-53(Fe)-TiO_2_ could eliminate around 98% of tetracycline after 150 min of illumination with a 300 W Xenon lamp. In another study, Gao, et al.^58^ indicated a poor photocatalytic performance for MIL-53(Fe) because of the fast recombination of electron–hole pairs. They overcame this problem by adding persulfate as an electron acceptor and obtained around 100% removal of acid orange 7 after 90 min of LED irradiation at the intensity of 0.47 mW/cm^2^.

**Figure 7:**
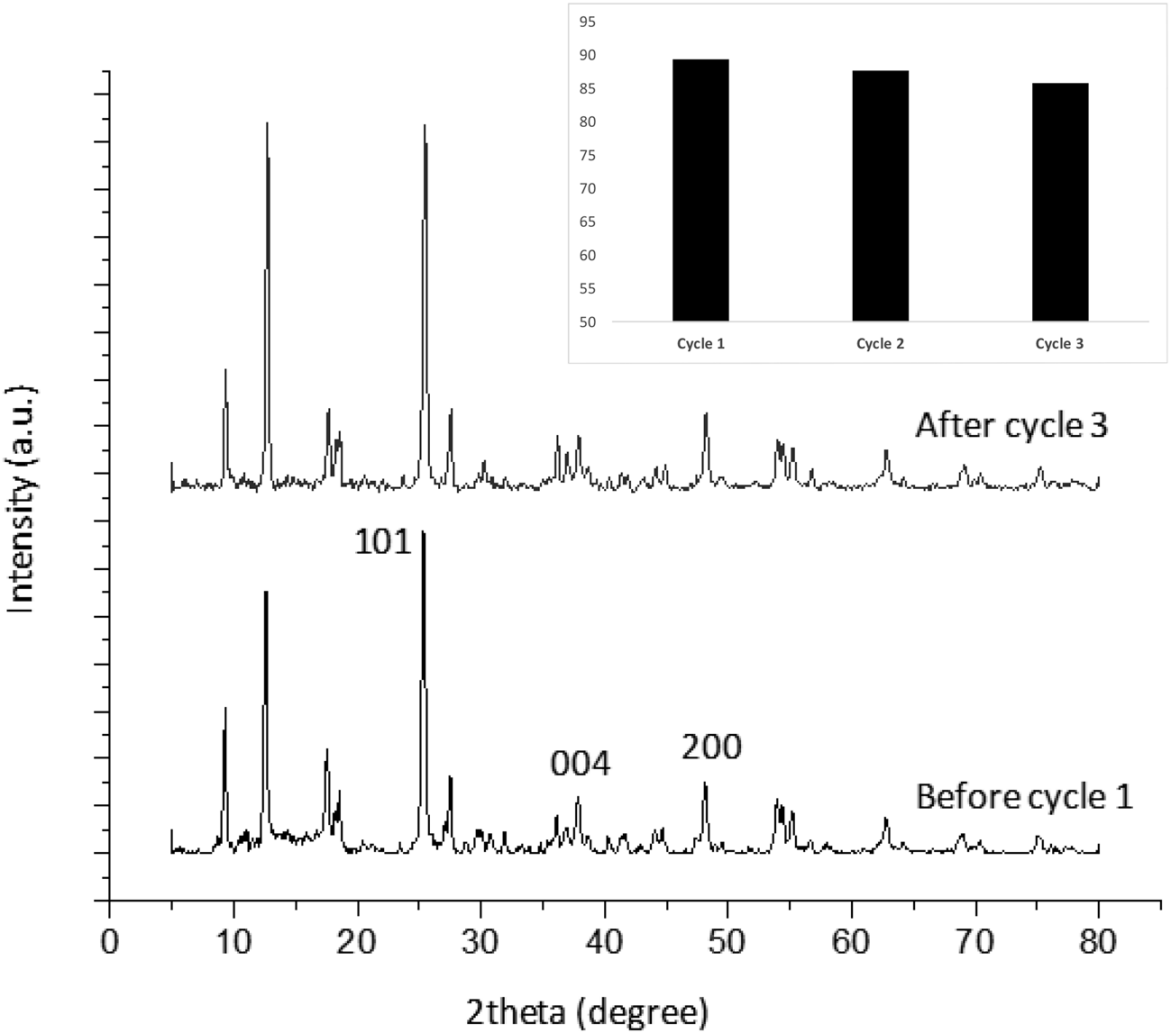
XRD patterns of fresh and recycled TiO_2_-MIL-53(Fe); the inset shows photocatalytic diazinon removal in three cycles of photocatalyst reuse.

Figure 7 depicts the data on the reusability and stability of TiO_2_-MIL-53(Fe). As the figure inset shows, the diazinon removal rate decreased from 89.35 to 85.8% after three cycles of photocatalyst reuse. The slight reduction in removal efficiency can be due to the loss of photocatalyst active sites during photocatalysis and regeneration. Moreover, the XRD patterns of the fresh and (three-cycle) reused TiO_2_-MIL-53(Fe) photocatalyst show the same peak patterns, confirming that the crystallized structure of TiO_2_-MIL-53(Fe) was not destroyed after three cycles of regeneration and reuse. In the XRD spectra, there are some distinctive peaks at 2θ of 9.31° and 12.61°, which agree with the previous study^33,59^. In addition, three other peaks at 2θ = 25.35°, 37.87°, and 48.13° could relate to the anatase structure of TiO_2_, corresponding to (101), (004), and (200) crystal planes, respectively^33,59,60^.

## Conclusion

In this study, based on fuzzy AHP-TOPSIS, availability emerged as the most important criterion for selecting photocatalysis systems, and TiO_2_-containing/Visible light was the best solution to implement in bench-scale studies of diazinon removal. We fabricated a TiO_2_-MIL-53(Fe) composite with a band gap of 1.8 eV and a low electron–hole recombination rate, which could remove diazinon at 89.35% after 180 min irradiation under LED visible light. However, we need more studies to optimize the conditions to improve the removal rate and shed light on other aspects of TiO_2_-MIL-53(Fe)/Visible light application, including mineralization efficiency and costs. As a limitation of the study, the alternatives were evaluated based on the experts' knowledge, experience, and local conditions, making the results not generalizable. Also, we found very few similar studies in the literature to compare the results for adequate discussion. Nevertheless, we demonstrated that MCDM techniques could be employed to select the best treatment alternative in the literature before designing experimental studies. Thus, researchers can integrate MCDM techniques into their systematic reviews to overcome the uncertainty in choosing a technology and design their original studies based on available evidence.

## Supplementary Information

Supplementary Information.

## Data Availability

All data used in this article are available for everyone upon reasonable request. To request the data from this study, you can contact Fatemeh.barjasteh@gmail.com.
