# Supplementary material for: Multi-criteria decision-making for prioritizing photocatalytic processes followed by TiO2-MIL-53(Fe) characterization and application for diazinon removal
Source: Sci Rep. 2023 May 1;13:7086. doi: 10.1038/s41598-023-34306-5 (PMC10150684; doi:10.1038/s41598-023-34306-5)
Supplement: Supplementary file 1 — Supplementary Information. [file 41598_2023_34306_MOESM1_ESM.docx]

Level 4

Alternatives

Level 3

Sub-Criteria

Level 2

Criteria

Level 1

Goal objective

**Fig. S1** Hierarchy structure for the selection of photocatalytic processes for diazinon removal from aqueous solutions


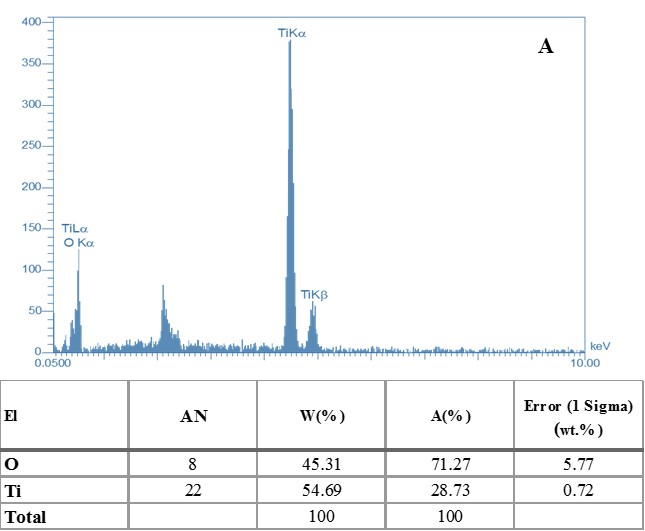

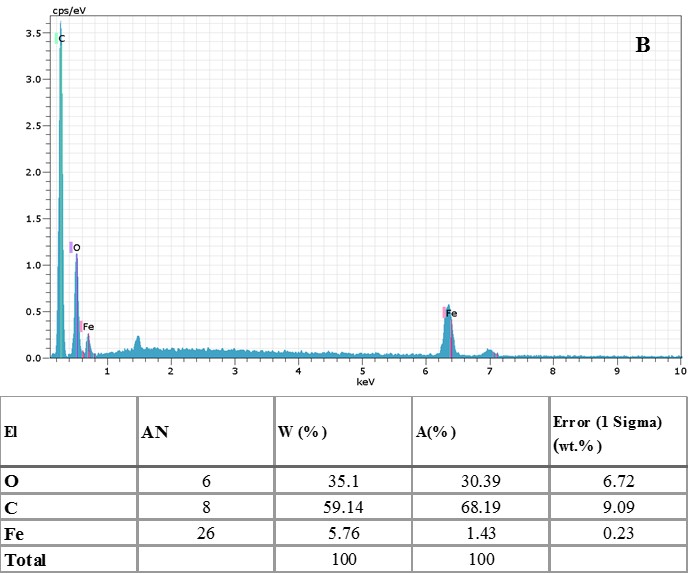


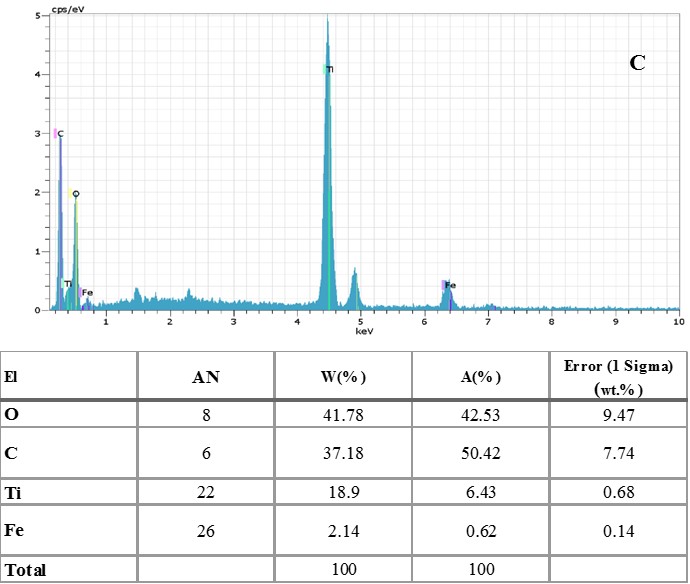


**Fig. S2** EDS plots and compositional analysis of A) TiO_2_, B) MIL-53(Fe), and C) TiO2-MIL-53(Fe)

**Table S1** Fuzzy numbers and linguistic variables used in AHP analysis

| Fuzzy number | Linguistic | Scale for Fuzzy number | | |
| --- | --- | --- | --- | --- |
|  |  | L | M | H |
| 1 | Equal | 0.5 | 1 | 2 |
| 2 | Weak | 1 | 2 | 3 |
| 3 | Not Bad | 2 | 3 | 4 |
| 4 | Preferable | 3 | 4 | 5 |
| 5 | Good | 4 | 5 | 6 |
| 6 | Fairly Good | 5 | 6 | 7 |
| 7 | Very Good | 6 | 7 | 8 |
| 8 | Absolute | 7 | 8 | 9 |
| 9 | Perfect | 8 | 9 | 9 |

| Fuzzy  number | Triangular Fuzzy number | | | Linguistic Variables |
| --- | --- | --- | --- | --- |
|  | **L** | **M** | **H** |  |
| 1 | 1 | 1 | 3 | Very Low (VL) |
| 3 | 1 | 3 | 5 | Low (L) |
| 5 | 3 | 5 | 7 | Medium (M) |
| 7 | 5 | 7 | 9 | High (H) |
| 9 | 7 | 9 | 9 | Very High (VH) |

**Table S2** Fuzzy sets for linguistic variables used in TOPSIS

**Table S3** Fuzzy geometric mean for criteria involved in AHP decision-making

C1: Removal Efficiency

C2: Cost

C3: Availability

C4: photocatalyst reusability

C5: Energy Consumption

C6: Safety

|  | **C1** | | | **C2** | | | **C3** | | | **C4** | | | **C5** | | | **C6** | | |
| --- | --- | --- | --- | --- | --- | --- | --- | --- | --- | --- | --- | --- | --- | --- | --- | --- | --- | --- |
|  | **L** | **M** | **U** | **L** | **M** | **U** | **L** | **M** | **U** | **L** | **M** | **U** | **L** | **M** | **U** | **L** | **M** | **U** |
| **C1** | 1 | 1 | 1 | 0.978 | 1.458 | 2.109 | 1.199 | 1.618 | 2.167 | 2.265 | 2.927 | 3.661 | 1.248 | 1.773 | 2.497 | 0.667 | 0.901 | 1.237 |
| **C2** | 0.474 | 0.685 | 1.022 | 1 | 1 | 1 | 1.022 | 1.458 | 2.018 | 1.854 | 2.688 | 3.661 | 1.237 | 1.954 | 2.931 | 0.667 | 0.895 | 1.210 |
| **C3** | 0.461 | 0.617 | 0.833 | 0.495 | 0.685 | 0.978 | 1 | 1 | 1 | 1.210 | 1.917 | 2.931 | 0.918 | 1.288 | 1.758 | 0.551 | 0.727 | 0.987 |
| **C4** | 0.273 | 0.341 | 0.441 | 0.273 | 0.372 | 0.539 | 0.341 | 0.521 | 0.521 | 1 | 1 | 1 | 0.506 | 0.776 | 1.210 | 0.323 | 0.412 | 0.556 |
| **C5** | 0.400 | 0.563 | 0.800 | 0.341 | 0.511 | 0.807 | 0.568 | 0.776 | 1.088 | 0.826 | 1.288 | 1.974 | 1 | 1 | 1 | 0.489 | 0.668 | 0.948 |
| **C6** | 0.807 | 1.109 | 1.498 | 0.826 | 1.116 | 1.498 | 1.013 | 1.375 | 1.814 | 1.797 | 2.423 | 3.091 | 1.054 | 1.496 | 2.044 | 1 | 1 | 1 |

**Table S4** Fuzzy geometric mean for sub-criteria involving in AHP decision-making

SUB 1:

|  | C11 | | | C12 | | |
| --- | --- | --- | --- | --- | --- | --- |
|  | **L** | **M** | **U** | **L** | **M** | **U** |
| C11 | 1 | 1 | 1 | 0.783 | 1.340 | 2.245 |
| C12 | 0.445 | 0.746 | 1.276 | 1 | 1 | 1 |

SUB 2:

|  | C21 | | | C22 | | |
| --- | --- | --- | --- | --- | --- | --- |
|  | **L** | **M** | **U** | **L** | **M** | **U** |
| C21 | 1 | 1 | 1 | 0.978 | 1.547 | 2.443 |
| C22 | 0.409 | 0.646 | 1.022 | 1 | 1 | 1 |

SUB 3:

|  | C61 | | | C62 | | |
| --- | --- | --- | --- | --- | --- | --- |
|  | **L** | **M** | **U** | **L** | **M** | **U** |
| C61 | 1 | 1 | 1 | 0.486 | 0.633 | 0.841 |
| C62 | 1.188 | 1.577 | 2.055 | 1 | 1 | 1 |

C11: Degradation efficiency C21: Material cost C61: Safety for personnel

C22: Equipment cost C12: Mineralization efficiency C62: Safety for the environment

**Table S5** Aggregate fuzzy decision matrix of TOPSIS

|  | C1 | | | C2 | | | C3 | | | C4 | | | C5 | | | C6 | | | C7 | | | C8 | | | C9 | | |
| --- | --- | --- | --- | --- | --- | --- | --- | --- | --- | --- | --- | --- | --- | --- | --- | --- | --- | --- | --- | --- | --- | --- | --- | --- | --- | --- | --- |
|  | **L** | **M** | **U** | **L** | **M** | **U** | **L** | **M** | **U** | **L** | **M** | **U** | **L** | **M** | **U** | **L** | **M** | **U** | **L** | **M** | **U** | **L** | **M** | **U** | **L** | **M** | **U** |
| TiO_2_-containing/UV | 5 | 7 | 9 | 1 | 5 | 9 | 1 | 3 | 5 | 7 | 9 | 9 | 3 | 6.4 | 9 | 5 | 7 | 9 | 5 | 7 | 9 | 1 | 3.8 | 7 | 1 | 5.4 | 9 |
| TiO_2_-containing /Vis | 5 | 7 | 9 | 3 | 6.2 | 9 | 1 | 3 | 5 | 1 | 1 | 3 | 3 | 6.4 | 9 | 5 | 7 | 9 | 3 | 5 | 7 | 1 | 7.6 | 9 | 1 | 5.8 | 9 |
| ZnO-containing /UV | 7 | 9 | 9 | 1 | 5 | 9 | 1 | 1 | 3 | 7 | 9 | 9 | 3 | 6.4 | 9 | 7 | 9 | 9 | 5 | 7 | 9 | 1 | 3.6 | 7 | 1 | 5.4 | 9 |
| ZnO-containing /Vis | 1 | 1 | 3 | 3 | 6.2 | 9 | 1 | 1 | 3 | 1 | 1 | 3 | 3 | 6.4 | 9 | 3 | 5 | 7 | 3 | 5 | 7 | 1 | 7.6 | 9 | 1 | 5.8 | 9 |
| WO_3_-containing /UV | 7 | 9 | 9 | 1 | 5.2 | 9 | 7 | 9 | 9 | 7 | 9 | 9 | 1 | 5.8 | 9 | 7 | 9 | 9 | 5 | 7 | 9 | 1 | 3.8 | 9 | 1 | 5.4 | 9 |

C1: Degradation efficiency C6: Photocatalyst reusability

C2: Mineralization efficiency C7: Energy consumption

C3: Material cost C8: Safety for personnel

C4: Equipment cost C9: Safety for the environment

C5: Availability

**Table S6** Normalized aggregate fuzzy decision matrix of TOPSIS

|  | C1 | | | C2 | | | C3 | | | | C4 | | | | C5 | | | | C6 | | | | C7 | | | | C8 | | | | C9 | | |
| --- | --- | --- | --- | --- | --- | --- | --- | --- | --- | --- | --- | --- | --- | --- | --- | --- | --- | --- | --- | --- | --- | --- | --- | --- | --- | --- | --- | --- | --- | --- | --- | --- | --- |
|  | **L** | **M** | **U** | **L** | **M** | **U** | **L** | **M** | **U** | **L** | | **M** | **U** | **L** | | **M** | **U** | **L** | | **M** | **U** | **L** | | **M** | **U** | **L** | | **M** | **U** | **L** | | **M** | **U** |
| TiO_2_/UV | 0.556 | 0.778 | 1.000 | 0.111 | 0.556 | 1.000 | 0.111 | 0.333 | 0.556 | 0.778 | | 1.000 | 1.000 | 0.333 | | 0.711 | 1.000 | 0.556 | | 0.778 | 1.000 | 0.556 | | 0.778 | 1.000 | 0.111 | | 0.422 | 0.778 | 0.111 | | 0.600 | 1.000 |
| TiO_2_/Vis | 0.556 | 0.778 | 1.000 | 0.333 | 0.689 | 1.000 | 0.111 | 0.333 | 0.556 | 0.111 | | 0.111 | 0.333 | 0.333 | | 0.711 | 1.000 | 0.556 | | 0.778 | 1.000 | 0.333 | | 0.556 | 0.778 | 0.111 | | 0.844 | 1.000 | 0.111 | | 0.644 | 1.000 |
| ZnO/UV | 0.778 | 1.000 | 1.000 | 0.111 | 0.556 | 1.000 | 0.111 | 0.111 | 0.333 | 0.778 | | 1.000 | 1.000 | 0.333 | | 0.711 | 1.000 | 0.778 | | 1.000 | 1.000 | 0.556 | | 0.778 | 1.000 | 0.111 | | 0.400 | 0.778 | 0.111 | | 0.600 | 1.000 |
| ZnO/Vis | 0.111 | 0.111 | 0.333 | 0.333 | 0.689 | 1.000 | 0.111 | 0.111 | 0.333 | 0.111 | | 0.111 | 0.333 | 0.333 | | 0.711 | 1.000 | 0.333 | | 0.556 | 0.778 | 0.333 | | 0.556 | 0.778 | 0.111 | | 0.844 | 1.000 | 0.111 | | 0.644 | 1.000 |
| WO_3_/UV | 0.778 | 1.000 | 1.000 | 0.111 | 0.578 | 1.000 | 0.778 | 1.000 | 1.000 | 0.778 | | 1.000 | 1.000 | 0.111 | | 0.644 | 1.000 | 0.778 | | 1.000 | 1.000 | 0.556 | | 0.778 | 1.000 | 0.111 | | 0.422 | 1.000 | 0.111 | | 0.600 | 1.000 |

C1: Degradation efficiency C6: Photocatalyst reusability

C2: Mineralization efficiency C7: Energy consumption

C3: Material cost C8: Safety for personnel

C4: Equipment cost C9: Safety for the environment

C5: Availability

|  | C1 | | | C2 | | | C3 | | | | C4 | | | | C5 | | | | C6 | | | | C7 | | | | C8 | | | | C9 | | |
| --- | --- | --- | --- | --- | --- | --- | --- | --- | --- | --- | --- | --- | --- | --- | --- | --- | --- | --- | --- | --- | --- | --- | --- | --- | --- | --- | --- | --- | --- | --- | --- | --- | --- |
|  | **L** | **M** | **U** | **L** | **M** | **U** | **L** | **M** | **U** | **L** | | **M** | **U** | **L** | | **M** | **U** | **L** | | **M** | **U** | **L** | | **M** | **U** | **L** | | **M** | **U** | **L** | | **M** | **U** |
| TiO_2_/UV | 0.043 | 0.104 | 0.203 | 0.006 | 0.056 | 0.164 | 0.008 | 0.041 | 0.104 | 0.033 | | 0.080 | 0.134 | 0.036 | | 0.106 | 0.199 | 0.033 | | 0.064 | 0.115 | 0.046 | | 0.093 | 0.167 | 0.006 | | 0.035 | 0.096 | 0.009 | | 0.078 | 0.182 |
| TiO_2_/Vis | 0.043 | 0.104 | 0.203 | 0.018 | 0.069 | 0.164 | 0.008 | 0.041 | 0.104 | 0.005 | | 0.009 | 0.045 | 0.036 | | 0.106 | 0.199 | 0.033 | | 0.064 | 0.115 | 0.028 | | 0.066 | 0.130 | 0.006 | | 0.070 | 0.124 | 0.009 | | 0.084 | 0.182 |
| ZnO/UV | 0.061 | 0.134 | 0.203 | 0.006 | 0.056 | 0.164 | 0.008 | 0.014 | 0.063 | 0.033 | | 0.080 | 0.134 | 0.036 | | 0.106 | 0.199 | 0.046 | | 0.082 | 0.115 | 0.046 | | 0.093 | 0.167 | 0.006 | | 0.033 | 0.096 | 0.009 | | 0.078 | 0.182 |
| ZnO/Vis | 0.009 | 0.015 | 0.068 | 0.018 | 0.069 | 0.164 | 0.008 | 0.014 | 0.063 | 0.005 | | 0.009 | 0.045 | 0.036 | | 0.106 | 0.199 | 0.020 | | 0.046 | 0.089 | 0.028 | | 0.066 | 0.130 | 0.006 | | 0.070 | 0.124 | 0.009 | | 0.084 | 0.182 |
| WO_3_/UV | 0.061 | 0.134 | 0.203 | 0.006 | 0.058 | 0.164 | 0.057 | 0.123 | 0.188 | 0.033 | | 0.080 | 0.134 | 0.012 | | 0.096 | 0.199 | 0.046 | | 0.082 | 0.115 | 0.046 | | 0.093 | 0.167 | 0.006 | | 0.035 | 0.124 | 0.009 | | 0.078 | 0.182 |

**Table S7** Weighted normalized fuzzy decision matrix of TOPSIS

C1: Degradation efficiency C6: Photocatalyst reusability

C2: Mineralization efficiency C7: Energy consumption

C3: Material cost C8: Safety for personnel

C4: Equipment cost C9: Safety for the environment

C5: Availability
